# Routine Screening Mammogram Leading to the Incidental Diagnosis of a Metastatic Neuroendocrine Breast Cancer (NEBC) from an Unrecognized Asymptomatic Small Bowel Neuroendocrine Tumor

**DOI:** 10.7759/cureus.23302

**Published:** 2022-03-18

**Authors:** Sura Alqaisi, Ali Rahman, Martin Barnes, William LiPera, Alan T Kaell

**Affiliations:** 1 Internal Medicine, Donald and Barbara Zucker School of Medicine at Hofstra/Northwell, Port Jefferson, USA; 2 Internal Medicine, Mather Hospital at Northwell Health, Port Jefferson, USA; 3 Hematology/Oncology, Stony Brook University Hospital, Port Jefferson, USA

**Keywords:** neuroendocrine breast tumor, somatostatin analogues, gastrointestinal carcinoid tumor, screening mammogram, chromogranin and synaptophysisn positive tumor

## Abstract

Neuroendocrine neoplasms (NENs) are epithelial neoplasms with predominant neuroendocrine differentiation that arise in the gastrointestinal tract, unique to the site of origin, such as the pancreas and small intestine. Neuroendocrine breast carcinoma (NEBC) is a rare tumor. Diagnosing NEBC is challenging because there is no specific clinical presentation, as it is usually presented as a breast lump. Therefore, diagnosing NEBC before biopsy is difficult. Another challenge in diagnosing NEBC is to know whether it is primary or metastatic. We present a case of a 60-year-old woman found to have a solid left breast nodule during routine screening mammography. Tissue biopsy was found to be consistent with metastatic NEBC. The patient was found to have primary small intestine asymptomatic NENs on further diagnostic tests. Eventually, she had a lumpectomy and started on lanreotide (Somatuline) intramuscular monthly injections.

As per literature, metastatic NEBC is infrequent. It was considered a poor prognostic breast tumor, as it is usually presented as hormonally negative breast cancer. Management of metastatic versus primary NEBC is still more controversial. Gastroenteropancreatic NENs are treated with long-acting somatostatin analogs with good prognostic results.

## Introduction

Neuroendocrine neoplasms (NENs) arise in the gastrointestinal tract, mainly in the pancreas or small intestine, with neuroendocrine differentiation [1,2]. Neuroendocrine breast carcinoma (NEBC) is a rare tumor that accounts for 0.27-0.5% of all NEN tumors and only 1% of breast cancer [1]. Usually, NENs are diagnosed by tissue biopsy, presenting as breast nodules. Deciding whether it is primary or metastatic NEBC will be based on the presence of other organs with NENs [1,3,4]. NEBC usually has no symptoms due to neuroendocrine tumor. Therefore, tissue biopsy is crucial in diagnosing NEBC. This case is about a patient who presented for routine mammography, found to have a breast lump. Biopsy indicated metastatic NEBC. The patient was found to have primary small bowel NENs on further imaging.

## Case presentation

**Figure 1:**
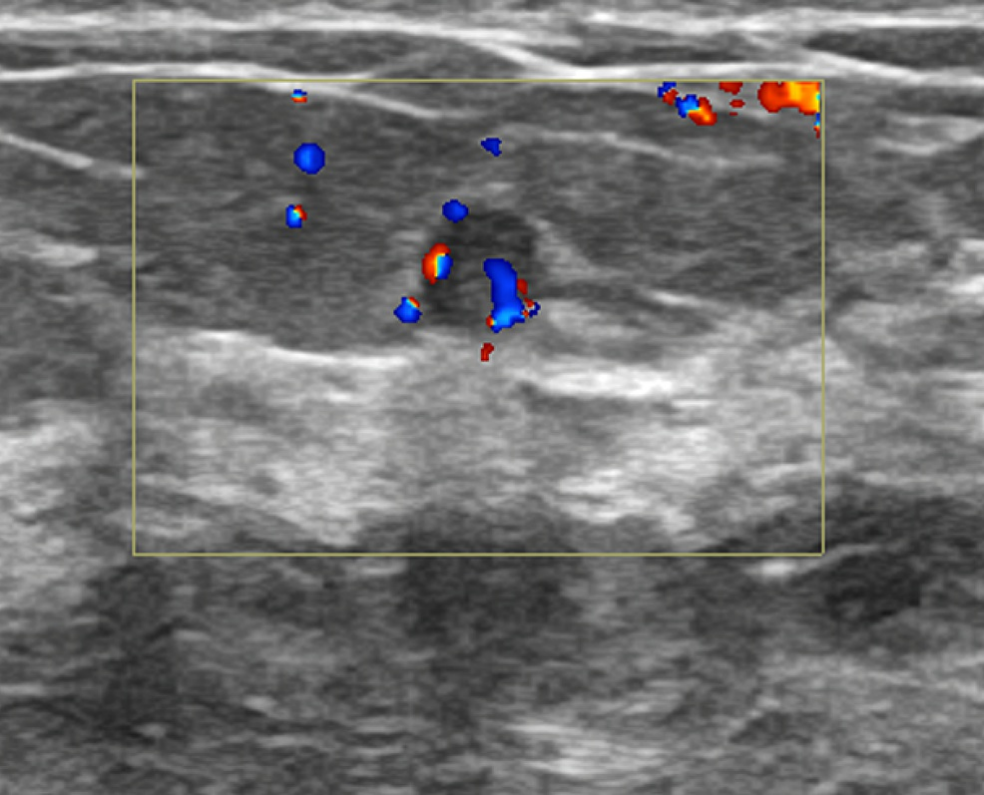
Ultrasound image of the breast demonstrating a hypoechoic solid lesion with posterior shadowing and associated internal vascularity

**Figure 2:**
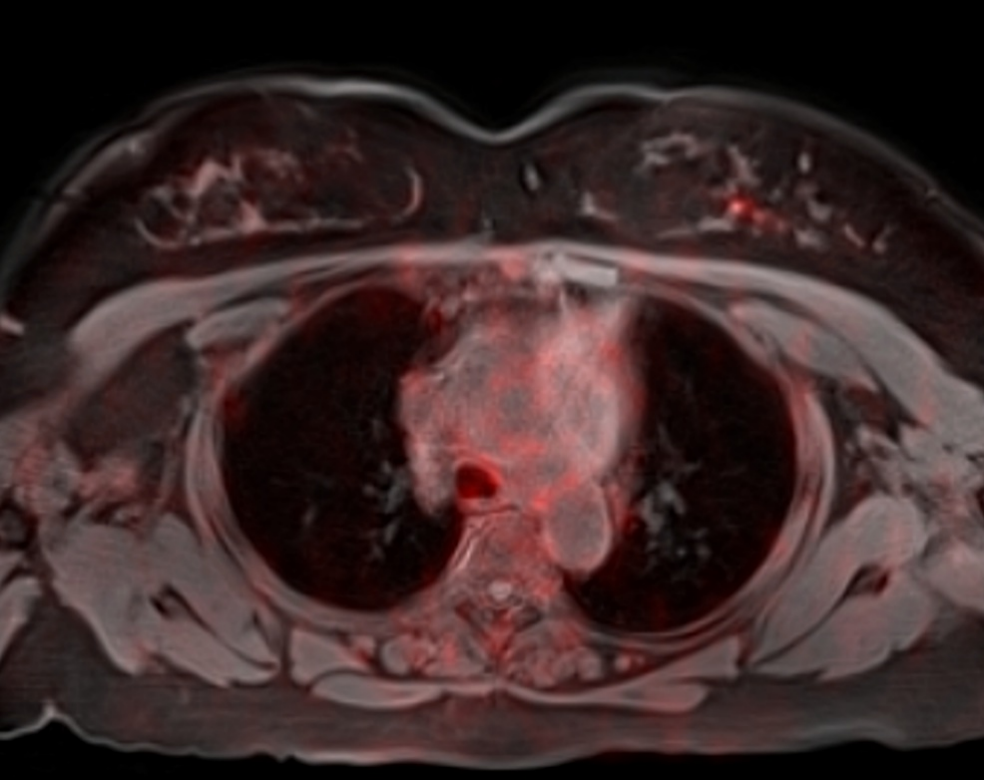
Fused axial image from gallium dotatate PET-MRI demonstrating focal radiotracer uptake in the left breast reflecting metastasis to the breast PET, positron emission tomography; MRI, magnetic resonance imaging.

**Figure 3:**
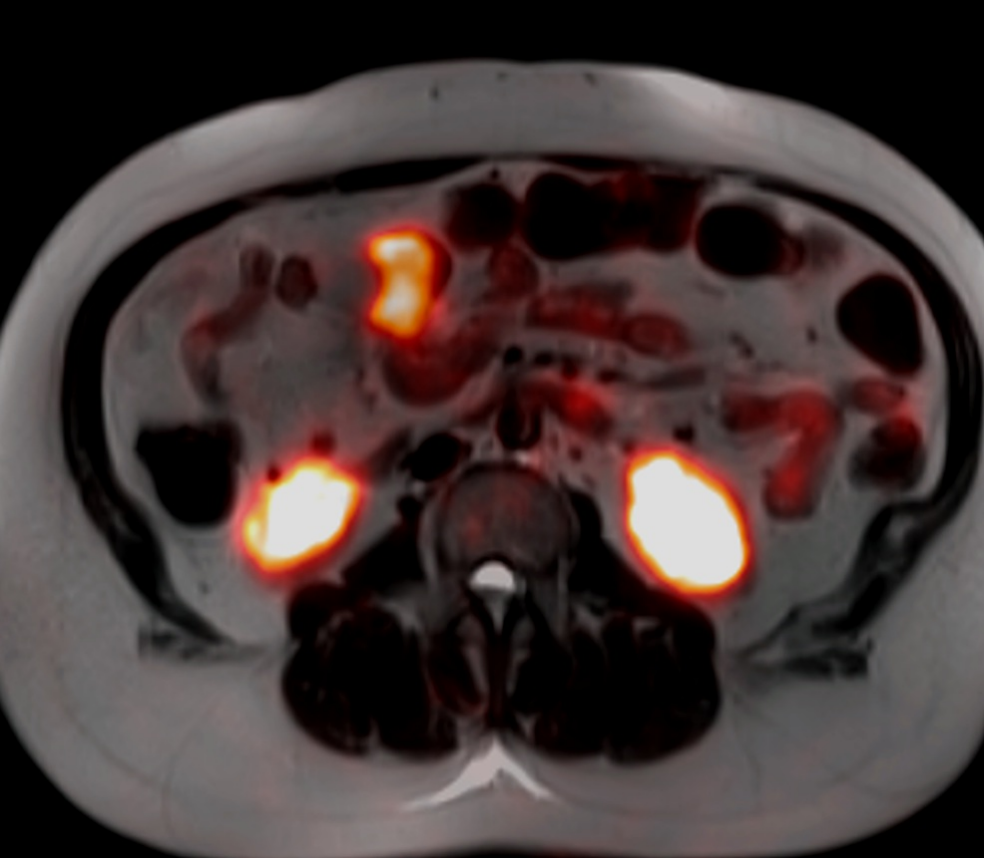
Fused axial image from gallium dotatate PET-MRI demonstrating focal radiotracer uptake associated with the loop of small bowel represent primary small bowel carcinoid PET, positron emission tomography; MRI, magnetic resonance imaging.

**Figure 4:**
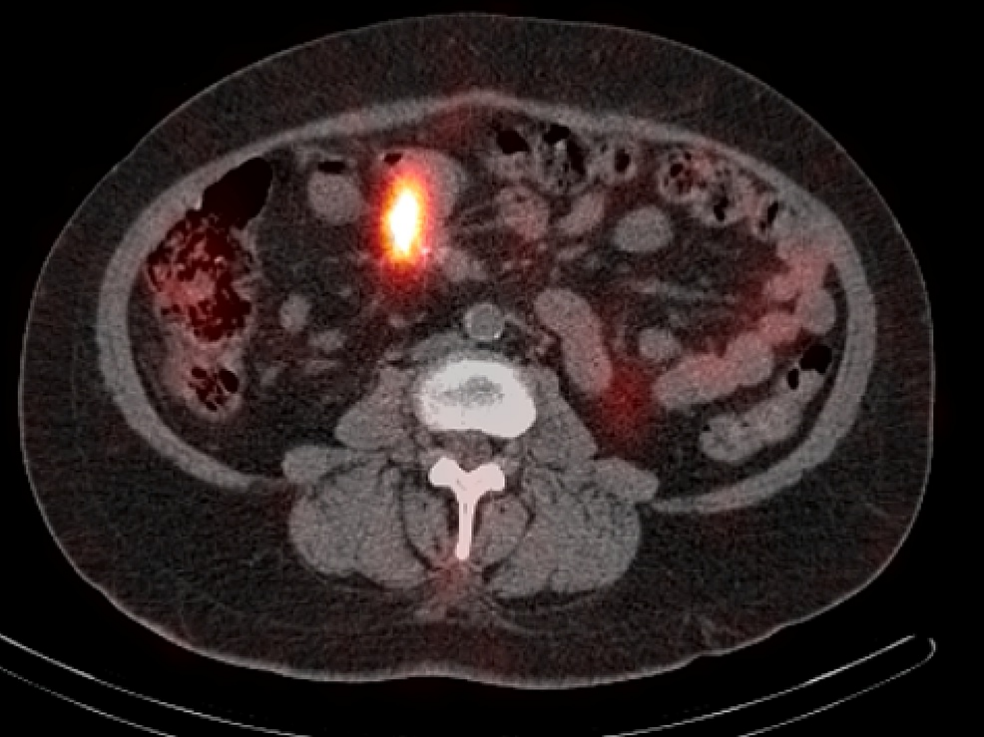
Fused octeriotide uptake scan with SPECT-CT demonstrating a focal radiotracer uptake associated with thickened small bowel lobe and adjacent mesenteric metastasis SPECT, single-photon emission computed tomography.

**Figure 5:**
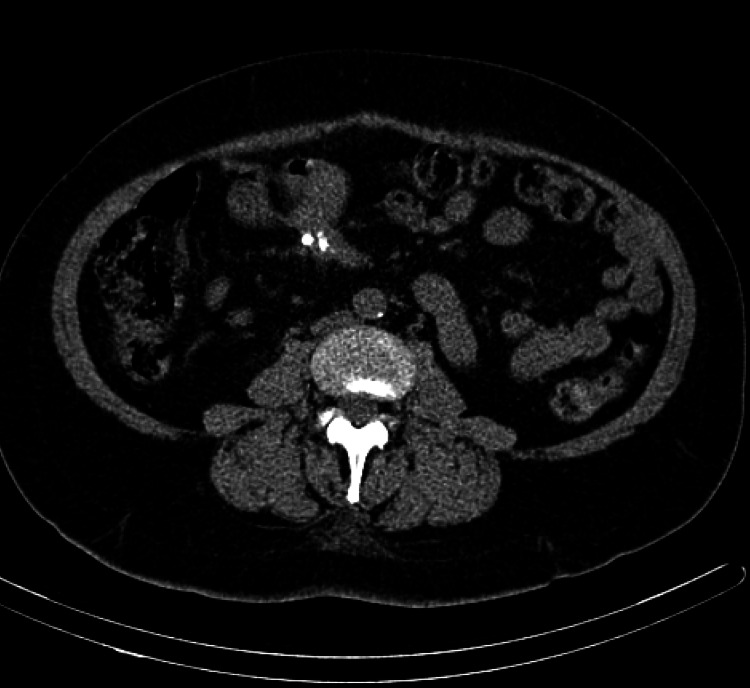
Axial CT without intravenous or enteric contrast demonstrating focal small bowel wall thickening with adjacent partially calcified mesenteric soft tissue lesion (this represents a mesenteric met with desmoplastic reaction from tumor secretion of serotonin) CT, computerized tomography.

A 60-year-old Caucasian woman was found to have a 4-mm solid nodule on her left breast during screening mammography (Figure 1). Then she had an ultrasound-guided core biopsy of her left breast mass, which showed a picture of a low-grade neuroendocrine tumor. She tested negative for estrogen and progesterone receptors, but was diffusely positive for chromogranin and synaptophysin. The patient's medical history is significant for autoimmune hepatitis, for which she consulted a hepatologist. The patient was then examined with abdominal and full-body imaging to look for possible primary NENs. On positron emission tomography/magnetic resonance (PET/MR) gallium scan, she was found to have prominent focal tracer uptake in the left breast, tracer activity corresponding to the thickening of the small bowel loop most consistent with the primary neuroendocrine tumor, and increased radiotracer uptake at the external inguinal chain LN, as shown in Figures 2-4. The patient denied any complaints; her blood tests were normal. Then she had surgical removal of the breast mass and started on Somatuline 120 mg monthly injections. The patient has been stable for over two years, with no complaints and no new growth based on her images in Figure 5.

## Discussion

NENs, usually present in the sixth or the seventh decade of life, arise mainly in the pancreas or the tubular gastrointestinal tract. Metastasis to the breast has been reported sporadically throughout the literature [1-3].

NEBC usually has no distinct presentation and is generally present as any other tumor as a solid mass [1,2]. Therefore, diagnosis of NEBC is not possible unless core or excisional biopsy with the typical expression of immunohistochemical markers is performed [1,2]. Another challenge is to know whether it is a primary NEBC or metastatic tumor because the treatment for the two is different. Primary NEBC is usually treated as invasive breast carcinoma [4].

To differentiate between primary versus metastatic NEBC, we have to look for the primary NEN, which is mostly present in the GI tract [1,3]. Thus, further imaging like MRI, PET scan, and PET/MR Ga68 is warranted.

The NEBC prognosis was considered poor, given that these tumors are estrogen receptor-negative [1,3]. However, implementing surgical resection and using long-acting somatostatin analog (SSA) have shown that NEBC has a better prognosis than other types of breast cancer, especially if it is detected early [4,5].

No proven effective chemotherapy regimen exists, but somatostatin receptors (SSTRs) are the preferred first-line option for NENs associated with several hormonal syndromes as well as for growth control in well-differentiated, unresectable, or metastatic gastroenteropancreatic NENs as in our case [4,5].

## Conclusions

NEBC, a rare type of primary or metastatic breast carcinoma, typically has no distinct clinical presentation. Biopsy and demonstration of classical markers are necessary for diagnosis. If discovered in a patient without a history of known primary NEN in another organ, imaging is warranted to seek asymptomatic and non-secreting tumors in the GI tract, pancreas, or elsewhere. Usually, the patient is started with MRI/magnetic resonance cholangiopancreatography (MRCP), and if negative, the treatment proceeds with octreotide scintigraphy that includes the detection and localization of a variety of neuroendocrine and other tumors as well as their metastases, the staging of patients with neuroendocrine tumors, and the follow-up of patients with known disease.
